# Papillary hyperplasia of the gallbladder diagnosed as gallbladder cancer before surgery: A case report

**DOI:** 10.1016/j.ijscr.2021.106542

**Published:** 2021-11-03

**Authors:** Shigetoshi Naito, Tomoaki Noritomi, Yoshihisa Fukuda, Yuko Goto, Takuro Hieda, Suguru Hasegawa

**Affiliations:** a Department of Surgery, Fukuoka Tokushukai Hospital, Fukuoka, Japan; b Department of Gastroenterology, Fukuoka Tokushukai Hospital, Fukuoka, Japan; c Department of Pathology, Fukuoka Tokushukai Hospital, Fukuoka, Japan; d Department of Gastroenterological Surgery, Faculty of Medicine, Fukuoka University, Fukuoka, Japan

**Keywords:** CT, computed tomography, EUS, endoscopic ultrasonography, FDG, fluorodeoxyglucose, FNA, fine needle aspiration, LC, laparoscopic cholecystectomy, MRI, magnetic resonance imaging, OC, open cholecystectomy, PBM, pancreaticobiliary maljunction, PET, positron emission tomography, Case report, Gallbladder, Papillary hyperplasia, Gallbladder cancer

## Abstract

**Introduction and importance:**

Gallbladder cancer has a poor prognosis. Therefore, an accurate diagnosis is required, for which various tests are performed. However, in some cases, it is difficult to distinguish between benign and malignant diseases before surgery. Papillary hyperplasia of the gallbladder is known for its secondary changes. Papillary hyperplasia of the gallbladder, which is known for its secondary changes, is a benign disease. We encountered papillary hyperplasia of the gallbladder with morphological changes over the course of 1 year. In addition, the tumor was suggested to be malignant during various examinations. We present a case of papillary hyperplasia of the gallbladder showing an increasing tendency and findings indicative of malignancy on imaging.

**Presentation of case:**

A 70-year-old man underwent routine abdominal ultrasonography every year. We observed that the gallbladder wall was thickened. The tumor size was 24 mm. FDG-PET and other examinations indicated malignancy requiring surgery.

**Clinical discussion:**

Accurate diagnosis of gallbladder tumor is difficult only by diagnostic imaging. There are problems with preoperative cytology and histology. FS can be an important test to avoid extended surgery.

**Conclusion:**

We report a rare case of papillary hyperplasia of the gallbladder, which was difficult to diagnose. Even when morphological changes and imaging findings suggest malignancy, similar findings could appear in papillary hyperplasia of the gallbladder owing to chronic inflammation.

## Highlights

• Gallbladder cancer has a poor prognosis.
• It is difficult to distinguish between benign diseases before surgery.
• Papillary hyperplasia of the gallbladder shows secondary changes.
• Papillary hyperplasia of the gallbladder can be confused for a malignancy.

## 1 Introduction

Gallbladder cancer is a disease with a poor prognosis [1]. Even with various examinations, diagnosis may be challenging. Therefore, when morphological findings suggesting changes over time or malignancy are confirmed, determination of malignancy and treatment should be considered. Here, we report the case of a patient with papillary hyperplasia of the gallbladder who was diagnosed with gallbladder cancer before surgery.

This work has been reported in line with the SCARE criteria [2].

## 2 Presentation of case

**Fig. 1:**
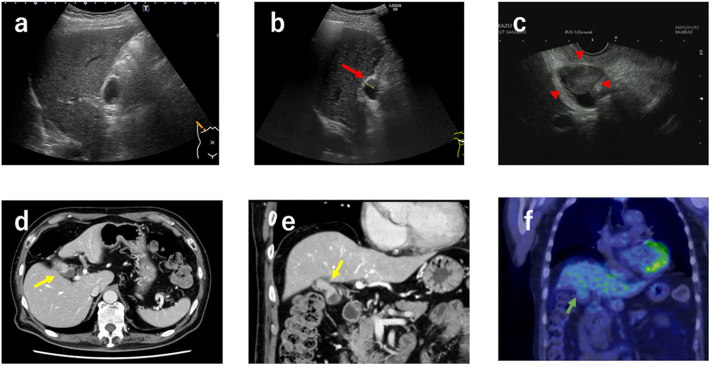
Preoperative transabdominal ultrasonography(a–b), endoscopic ultrasonography (EUS) (c), computed tomography (CT) (d), EUS (e), and positron emission tomography (PET)-CT(f). (a) Previous year's transabdominal ultrasonography finding was normal. (b) In this US, wall thickening is noted (red arrow). (c) Endoscopic ultrasonography reveals subpedunculated, broad-based, elevated lesions with irregular surfaces (red arrow head). (d–e) A tumor is found that protruded into the lumen of the bottom of the gallbladder in the contrast-enhanced (yellow arrow) CT. (f) PET-CT shows mild uptake of fluorodeoxyglucose (FDG) in the tumor (green arrow). (For interpretation of the references to colour in this figure legend, the reader is referred to the web version of this article.)

A 70-year-old man regularly visited our hospital for follow-up of diabetes, stroke, myocardial infarction, and renal artery stenosis. Regular abdominal ultrasonography was routinely performed annually to assess renal artery blood flow. We observed that the gallbladder wall, which had been normal until this time (Fig. 1a), was thickened. No abnormalities were found, particularly in the thoracoabdominal region. The carcinoembryonic antigen and carbohydrate antigen 19–9 levels were within the normal range (<37 and <5.0 U/mL, respectively). Abdominal ultrasonography revealed hypoechoic elevated lesions and wall thickening in the body of the gallbladder (Fig. 1b). Endoscopic ultrasonography (EUS) revealed subpedunculated, broad-based, elevated lesions with irregular surfaces. The echo band suggesting the muscular layer at this site was interrupted, and serosal infiltration was suspected (Fig. 1c). Contrast-enhanced computed tomography (CT) revealed a tumor that protruded into the lumen of the bottom of the gallbladder (Fig. 1d and e). The tumor size was 24 mm, with a contrast effect. Magnetic resonance imaging (MRI) also revealed polypoid elevated lesions. Pancreaticobiliary maljunction (PBM) were not observed. Positron emission tomography (PET)-CT showed mild uptake of fluorodeoxyglucose (FDG) in the tumor (maximum standard uptake value, 2.98) (Fig. 1f). The tumor progressed on routine abdominal examinations. Moreover, pedunculated polyps >1 cm are considered cancerous. Therefore, the patient was diagnosed with gallbladder cancer.

He refused additional surgery and demanded one-step radical resection. Due to the risk of advanced cancer, hepatectomy of segments 4a and 5 with bile duct resection was planned. Regarding intraoperative findings, the gallbladder was hard and atrophied, adhering to the surrounding omentum. The areas from the gallbladder neck to the cystic duct were also hard, and the border was unclear; therefore, it was judged that simple cholecystectomy was extremely difficult. To secure a sufficient margin, a part of the omentum was attached to the gallbladder, and the surgery was completed as hepatectomy of segments 4a and 5 with bile duct resection as scheduled.

**Fig. 2:**
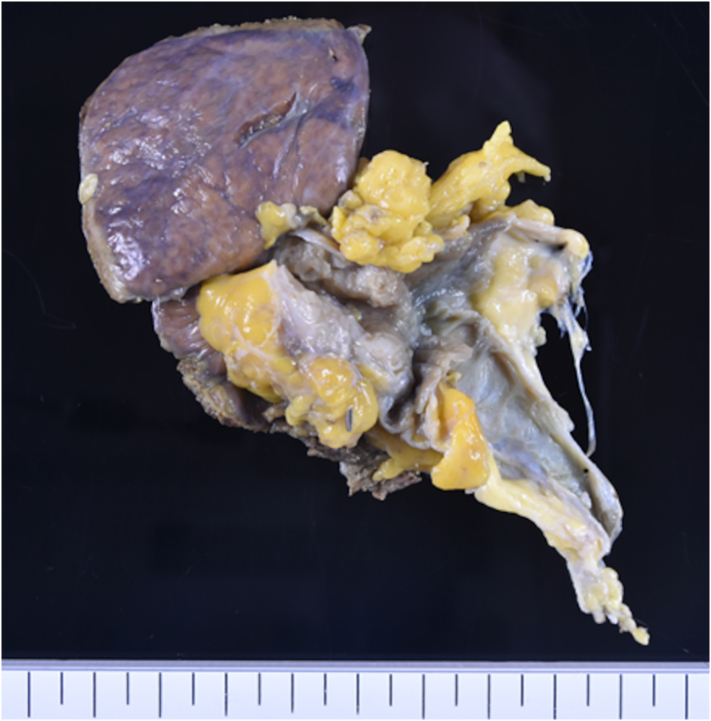
Macroscopic findings of the resected specimen. A surface irregular mucosa is observed on the wall on the bile duct side of adenomyomatosis.

**Fig. 3:**
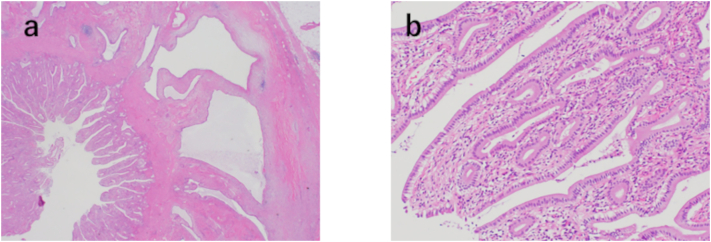
Histopathological examination (a–b). (a) Hematoxylin and eosin (H&E) staining for the site where the mucosa with irregular surface. The magnification of the micrograph is 40×. (b) Higher magnification (200×). (a) Papillary epithelium is growing in the gallbladder. (b) The mucosa is observed with no mitotic figures or atypia, and it is judged that it is not a malignant disease.

On pathological observation, the wall at the bottom of the gallbladder appeared thickened, with cystic lesions. These cystic lesions were considered to be adenomyomatosis. In addition, a surface irregular mucosa was observed on the wall of the bile duct side of adenomyomatosis (Fig. 2). Histopathological examination revealed papillary epithelium in the areas of the mucosa with irregular surfaces. No mitotic figure or atypia was noted at that site, and malignancy was refuted (Fig. 3a and b). Finally, we diagnosed the tumor as papillary hyperplasia of the gallbladder. The patient was discharged 14 days postoperatively without complications. He is doing well without any complications since 6 months following the surgery.

## 3 Discussion

Papillary hyperplasia of the gallbladder is a benign disease; however, its diagnosis is challenging, and performing surgery for diagnostic purposes is not uncommon. In the current case, morphological changes occurred during follow-up, and FDG-PET and other examinations indicated malignancy requiring surgery. To the best of our knowledge, papillary hyperplasia of the gallbladder, which causes morphological changes and was observed during abdominal ultrasonography, was not found in previous reports. In previous studies, some cases were found accidentally. This current case confirms that papillary hyperplasia of the gallbladder can cause dramatic changes in 1 year.

Papillary hyperplasia of the gallbladder is known as secondary mucosa degeneration related to chronic inflammatory diseases [3]. In cholelithiasis, hyperplastic changes could occur regardless of infection. PBM is considered a cause other than cholelithiasis [3], [4]. The reflux of the pancreatic juice into the bile duct is associated with chronic inflammatory changes. This phenomenon is caused by the activation of bile acids and pancreatic enzymes [5]. In addition, papillary hyperplasia of the gallbladder caused by ulcerative colitis, primary sclerosing cholangitis, and tuberculosis has been reported [6], [7], [8]. However, in this case, no stones, PBM, and obvious systemic inflammatory disease could be confirmed. The cause of chronic inflammation is unknown, and there may have been a PBM that could not be identified by MRI. Alternatively, Rokitansky–Aschoff sinus could have segmented the gallbladder, causing obstruction and chronic inflammation in the periphery. Previous reports have revealed that segmental adenomyomatosis causes cholestasis and is associated with cholelithiasis and cholecystitis [9].

In this case, it was difficult to distinguish it from cancer. If the elevated lesion of the gallbladder was 10 mm or more and it progressed into a solid hypoechoic and hypertrophied mass, the possibility of cancer could be ascertained [10], [11], [12], [13]. Nearly all conditions were met, and the tumor was judged to be cancerous. Contrast-enhanced CT and EUS examinations were performed to further improve the diagnostic accuracy.^11^ Kim et al. reported that multi-dissection CT provided 83.9% accuracy in the diagnosis of the local extent of gallbladder carcinomas, thereby demonstrating acceptable sensitivity and specificity [14]. Moreover, Azuma et al. reported that 86.5% of polypoid lesions were precisely diagnosed by EUS. The sensitivity, specificity, and positive and negative predictive values of EUS for the diagnosis of carcinoma were 91.7%, 87.7%, 75.9%, and 96.6%, respectively [15]. FDG-PET CT showed FDG uptake, which was suspected to be malignant. In this case, wall thickening due to adenomyomatosis and chronic inflammation could have made diagnostic imaging difficult. In addition, primary papillary hyperplasia of the gallbladder PBM cannot be detected easily by radiologic or other alternative examinations in practice [3]. We need to examine more cases of papillary hyperplasia of the gallbladder to define its characteristics for future diagnosis.

Since there was a possibility of malignancy in the preoperative diagnostic imaging, the surgical procedure, which would be a complete treatment even if the pathology turns out to be malignant, was decided in consultation with the patient. Extrahepatic bile duct resection and hepatic S4a + S5 resection were performed since the depth of invasion and extent of the tumor to the neck could not be determined. There are various reports on preoperative cytological diagnosis to avoid over-invasiveness. The results of the cytology and histology of gallbladder and bile duct lesions by endoscopic ultrasound-guided fine-needle aspiration (EUS-FNA) were reportedly good and no complications observed [16]. However, another study has reported that bile aspiration by EUS-FNA causes bile peritonitis due to bile leakage [17]. Therefore, FNA may impair curability in cases with possible surgical treatment and is not recommended. If malignancy is suspected but difficult to diagnose, surgery may be necessary for diagnostic and therapeutic purposes. Laparoscopic surgery is commonly performed as one of the minimally invasive treatments. Recent reports have shown that for accidental gallbladder cancer, adding curative surgery between the two groups, laparoscopic cholecystectomy (LC) and open cholecystectomy (OC), does not make a significant difference in surgical results [18], [19]. There are also reports that it is useful for confirming the diagnosis and deciding the treatment policy [18]. However, when LC is indicated for gallbladder cancer, port site recurrence and peritoneal recurrence as a result of bile leakage into the abdominal cavity due to gallbladder injury may be major problems [20]. Some reports have shown that the incidence of port site recurrence and peritoneal recurrence is as high as 10%–18% in accidental gallbladder cancer detected after LC [21], [22], [23], [24]. Therefore, we consider that OC should be performed for patients suspected of having gallbladder cancer. In addition, the patient had hoped to avoid two-stage surgery. Radical resection of gallbladder cancer is a hepatectomy with lymph node dissection. Extrahepatic bile duct resection is also used to prevent postoperative bile duct stenosis associated with cystic duct infiltration or lymph node dissection. However, this case was benign. Thus, our surgical procedure may have been too aggressive. This unusual gallbladder tumor resulted in performing extended surgery. Careful measures should be undertaken considering intraoperative frozen section diagnosis to avoid unnecessary extended surgical procedure for undiagnosed gallbladder tumor. Deng et al. reported that intraoperative frozen section analysis had a diagnostic accuracy of 93% in xanthogranulomatous cholecystitis [25]. Frozen section diagnosis can be a good diagnostic tool.

## 4 Conclusion

Papillary hyperplasia of the gallbladder is generally associated with chronic inflammation. However, this case had no gallstones or PBM and could have progressed rapidly in 1 year, and the patient was suspected to have gallbladder cancer on various imaging. The possibility of malignant disease was undeniable, and surgery was recommended based on the malignant disease. Surgeons should include not only cancer in the elevated lesions of the gallbladder with morphological changes, but also papillary hyperplasia of the gallbladder as a differential disease. In addition, aggressive intraoperative induction of frozen section diagnosis may prevent extended surgery of preoperatively undiagnosed gallbladder masses and lead to appropriate surgical procedures.

## Sources of funding

No special funding source was used to support this study.

## Ethical approval

The publication of this case was approved by the Ethics Committee of the Ome Fukuoka Tokushukai Hospital.

## Consent

The patient provided informed consent for publication of the data.

## Author contribution

SN performed the procedure as an operator and was a major contributor to the writing of the manuscript. YG performed the histological examinations. YF was in charge of various examinations for the patient. TH has acquired the data.TN and SH reviewed and contributed to the discussion and critical review of the draft versions of the manuscript. All authors read and approved the final manuscript.

## Registration of research studies

The current case report is not ‘First in Man’ studies.

## Guarantor

The Guarantor is Shigetoshi Naito and Tomoaki Noritomi.

## Declaration of competing interest

The authors declare that they have no competing interests.
